# Effectiveness of turmeric-enriched pellets to improve the immunity of *Clarias batrachus* toward motile *Aeromonas *septicemia disease

**DOI:** 10.12688/f1000research.28260.1

**Published:** 2021-03-02

**Authors:** Morina Riauwaty, Yusni I. Siregar, Isma Mulyani

**Affiliations:** 1 Fisheries and Marine Science Faculty, Universitas Riau, Pekanbaru, Riau, Indonesia

**Keywords:** Aeromoniasis, catfish, clinical sign, MAS diseases, self-defense

## Abstract

**Background**: Turmeric is known as a natural remedy to improve the immunity of organisms. This study aims to understand the effectiveness of turmeric-enriched pellets to improve the immunity of *Clarias batrachus *to *Aeromonas hydrophila*.

**Methods:** The study was conducted from May to August 2020. *C. batrachus *fingerlings, 7-8 cm total length (TL) and 4-5 g (BW) at baseline, were kept in 30 L aquaria (10 fishes/aquarium; three replicated/treatment). Commercial pellets were mixed with turmeric powder. There were five treatment groups: P0 (control, no turmeric); P1 (0.5 g turmeric per Kg of pellets); P2 (0.7 g/Kg); P3 (0.9 g/Kg); Pp (positive control). Thirty days after being feed with turmeric-enriched pellets, all groups of fish were infected with 0.1 ml (10 ^8^) of *A. hydrophila* suspension, intramuscularly. The P0 group did not receive injection, while Pp group were not fed with turmeric-enriched pellets but were infected with the bacteria. Fourteen days after infection, clinical signs and hematology of the fish were studied.

**Results:** Pp fish showed heavy clinical signs of *A. hydrophila* *,* such as loss of balance, pigmentation, hemorrhages and ulcers. P0 fish did not show any symptoms, while the treated fish reveled some clinical signs of *A. hydrophila* to a lesser extent than Pp, indicating that the fish is able to face the *A. hydrophila* attack. Hematology for Pp fish revealed high white blood cells, indicating that the fish were infected. The blood condition of the P0 fish, as well as those of the turmeric-treated fish were normal. In general, the P3 fish showed the least clinical signs of *A. hydrophila *and normal blood condition, indicating that P3 treatment is best.

**Conclusion:** The best turmeric dosage to improve the immunity of *C. batrachus* toward *A* *. hydrophila* infection is 0.9 g/Kg pellets.

## Introduction

*Clarias batrachus* or the catfish is a favored fish in Riau Province, Indonesia, due to its high economic value and high protein content. The demand of this fish is high, leading to the community culturing the fish, at large and household scale.

*C. batrachus* is relatively easy to be cultured. It is able to live in fair quality water and consumes a wide range of feed, including commercial pellets, food remains, and fish or chicken remains. This fish grows quickly and achieves marketable size, around 125 grams, within two months. However, this fish is vulnerable toward *Aeromonas hydrophila* attack, which causes motile *Aeromonas* septicemia (MAS) disease. This disease may cause mass death in fish or cause ulcers and hemorrhage in fish skin. Fish that suffer from the disease may die or be unmarketable, and this problem causes great loss in fish culture ^1^. 

So far, MAS disease is commonly prevented or cured using antibiotics. The use of antibiotics, however, has negative impacts as its residues may stay in fish flesh and endanger the health of consumers ^2^. Another alternative in preventing MAS disease in fish is by improving the immunity of the fish using natural remedies, such as turmeric. The root of turmeric contains natural materials, namely curcumin that is antibacterial and has immune-modulatory agents ^1^. The chemical components of turmeric are curcumin (diferuloylmethane), desmethoxycurcumin, and bisdemethoxycurcumin ^3^.

Turmeric is well known as a traditional remedy for humans and it has been widely used for its antimicrobial, anti-inflammatory, antioxidant properties, as a detoxification of toxins and is able to increase the immune system against disease ^2^. Therefore, turmeric has been used to improve fish health through immersing method ^4^. Unfortunately, that method is not very effective as the treated fish became stressed and it is not practical for large scale fish culture. Turmeric is not poisonous, and it can be consumed, but feeding fish with turmeric-enriched pellets has never been studied. To understand the effectiveness of turmeric-enriched pellets to improve the immunity of fish to *A. hydrophila*, this study aimed to assess the effectiveness of turmeric-enriched pellets in improving the immunity of *C. batrachus* towards *A. hydrophila*. 

## Methods

### Study design and fish

This research was conducted from May to August 2020 at the Parasite and Fish Diseases Laboratory, Aquaculture, Fisheries and Marine Science Faculty, Riau University. The experiments were carried out within the ethical guidelines provided by the research institution and national or international regulations.

*C. batrachus* fingerlings were obtained from the hatchery of the Riau Province’s Marine Fisheries and Department in Tibun, Pekanbaru. Fish chosen were actively swimming with no wounds or parasites. They were approximately 7–8 cm total length (TL) and 4–5 g body weight (BW) . The fish were reared in aquaria (30×40×40cm ^3^; 10 fish/aquarium) with aerators and filters. Prior to the treatment, the fish were acclimated to the laboratory environment for four days. A total of 150 fish (30 fish per treatment for five treatments). Blood samples were taken from 3 fish/treatment (total 15 fish).

### Experimental design

A completely randomized design with five treatment groups (three replications per treatment) was used in this research. The aquaria were grouped based on the turmeric treatments and in each group the aquaria were placed randomly based on lottery method. The treatments applied are as follows:

- P0 = negative control, no turmeric feed, no infection
- Pp = positive control, no turmeric feed, infected with *A. hydrophila*
- P1 = 0.5g turmeric in 1 Kg feed, infected with *A. hydrophila*
- P2 = 0.7g turmeric in 1 Kg feed, infected with *A. hydrophila*
- P3 = 0.9g turmeric in 1 Kg feed, infected with *A. hydrophila*

### Turmeric-enriched pellet preparation

Turmeric powder was made by slicing the turmeric, drying and grinding it using a blender. During the research, the fish were feed with commercial fish feed pellet (F999 with 35% protein content from the PT Central Proteina Prima Tbk). The powder was then mixed with fish feed pellets before the feed was given. The turmeric used in this study was obtained from the local market in Delima Street Pekanbaru and the turmeric was planted by local farmer. During the study the fish were fed *ad libitum*. The turmeric was mixed with a spoon of water and then mixed well with 1 Kg of pellets. The enriched pellet was then directly given to the fish, three times per day (morning, noon and afternoon).

On the 30th day, the fish were infected with *A. hydrophila* bacteria (intramuscularly 0.1 ml with a bacterial density of 1.0x108 CFU/mL). Prior to injection, the fish was sedated using clove oil, approximately 0.25 ml or 5 drops/L fresh water. The fish was put in the clove mixture for around 3 minutes until it shown inactive movement. After the injection the fish was returned to the rearing tank. 

After being infected, clinical sign of MAS disease (namely rotting of the tail, increased respiration rate and swollen abdomen, exophthalmia and lethargy) was monitored every day. By the 45th day of the experiment (14th days after infection) the blood condition of fish was studied.

Blood parameters were conducted two times, at baseline and in the 8 ^th^ week (end of the research period). Three fish from each aquaria were taken and their blood were obtained; fish were anesthetized using clove oil (5 drops/L) and blood was taken from the caudal vein, by inserting an EDTA (Merck) 10% wet syringe. Blood samples was kept in EDTA moistened vials, in a cool box filled with crushed ice. Total erythrocytes and leukocytes were counted using a Neubauer hemocytometer and then were calculated ^3^ and analyzed ^4^. Hematocrite and leucocrite levels were determined using heparinized micro-hematocrit capillaries that was centrifuged at 12,000 rpm for 3 minutes. Hemoglobin content in blood was measured using Sahli method ^5^.

### Data analysis

The parameters studied are as follows:

1. **Survival rate:** survival of the fish was monitored every day and data obtained were analyzed using ANOVA.
2. **Growth:** growth of fish was monitored one per week and data obtained were analyzed using ANOVA.
3. **Clinical signs:** after being treated with turmeric enriched pellet for 30 days, the fish was injected with *A hydrophyla* and the clinical sign of the MAS diseases in fish was monitored everyday (started at the injection day) for 7 days. Data obtained were described.
4. *Hematological condition:* Blood samples were taken 2 times. The first samples were taken prior to turmeric enriched pellet treatment. The second blood sampling was conducted in the 45 ^th^ day (14 days after the fish being infected with *A hydrophyla*). Hematological parameters measured were total erythrocyte, hematocrit levels, hemoglobin, total leucocyte and leucocyte differentiation. Data obtained were then described.

Data were analyzed using Microsoft Excel program for creating graphics and SPSS program to calculate the ANOVA of growth data.

## Results

### Survival

The survival of the fish various among treatment groups. After being fed with turmeric-enriched pellets for 30 days, the survival of the fish was 100% in all treatments. After infection, however it is clear that the survival rate of the infected fish decreased. In P0 group (no turmeric feed nor *A. hydrophila* infection), the survival rate was 100%. In contrast, in the Pp group (no turmeric with *A. hydrophila* infection), the survival of the fish by the end of the experiment was low (43.33%). The survival rate of *C. batrachus* for all groups is presented in Table 1.

**Table 1.** Survival rate of *Clarias batrachus* after being fed a turmeric-enriched feed and infected with *Aeromonas hydrophila.*

Treatments	Survival rate (%)	
	Day 0	Day 45
P0	100	100.00 ± 0.00 ^d^
Pp	100	43.33 ± 5.77 ^a^
P1	100	50.00 ± 10.00 ^a^
P2	100	66.66 ± 5.77 ^b^
P3	100	80.00 ± 10.00 ^d^

P0, control (no turmeric/not infected); Pp, positive control (no turmeric/infected); P1, 0.5g turmeric in 1 Kg feed and infected; P2, 0.7g/Kg and infected; P3, 0.9g/Kg and infected. Mean with standard error followed by different letters are significantly different (P<0.05)

All infected fish showed various clinical signs of MAS disease, namely ulcers, hemorrhage, pigmentation, swollen abdomen and eroded fins. MAS signs worsened as the turmeric dose was reduced: P1, rotting of the tail, increased respiration rate and swollen abdomen; P2, exophthalmia and lethargy; P3, clinical symptoms were unclear.

### Growth

**Figure 1.**
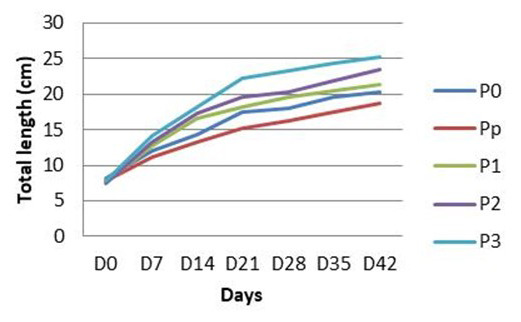
Total length of *Clarias batrachus* fed with turmeric enriched pellets and infected with *Aeromonas hydrophila* (on day 30). Po, control (no turmeric/not infected); Pp, positive control (no turmeric/infected); P1, 0.5g turmeric in 1 Kg feed and infected; P2, 0.7g/Kg and infected; P3, 0.9g/Kg and infected.

The growth pattern of *C. batrachus* is presented in Table 2. In general, the growth of fish in all treatment groups showed a similar pattern: they gained length throughout the experiment. However, by the end of the experiment, TL varied between groups. Fish that were treated with turmeric shown better growth than that of fish that do not receive any turmeric. The growth of fish reduced as the turmeric dosages decreased ( Table 2; Figure 1). 

**Table 2.** Total length of *Clarias batrachus* fed with turmeric enriched pellets and infected with *Aeromonas hydrophila* (on day 30).

Treatments	Total length (cm) per week							Daily growth rate (cm/day)
	D0	D7	D14	D21	D28	D35	D42	
P0	8.11	12.06	14.34	17.45	18.02	19.67	20.23	0.29
Pp	7.80	11.15	13.24	15.24	16.33	17.46	18.65	0.26
P1	7.60	12.67	16.56	18.12	19.56	20.56	21.37	0.33
P2	7.53	13.24	17.28	19.60	20.34	21.89	23.45	0.38
P3	7.82	14.14	18.22	22.21	23.23	24.43	25.23	0.41

Po, control (no turmeric/not infected); Pp, positive control (no turmeric/infected); P1, 0.5g turmeric in 1 Kg feed and infected; P2, 0.7g/Kg and infected; P3, 0.9g/Kg and infected.

**Figure 2.**
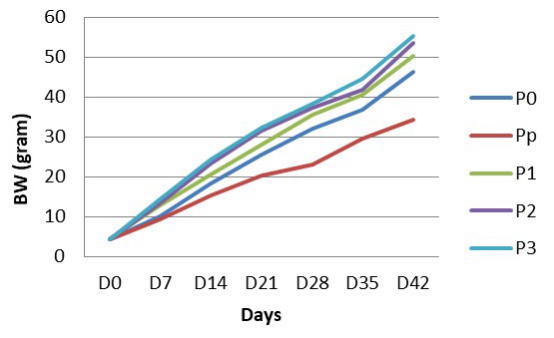
Body weight of *Clarias batrachus* fed with turmeric enriched pellets and infected with *Aeromonas hydrophila* (on day 30). P0, control (no turmeric/not infected); Pp, positive control (no turmeric/infected); P1, 0.5g turmeric in 1 Kg feed and infected; P2, 0.7g/Kg and infected; P3, 0.9g/Kg and infected.

As well as body length, BW of the treated fish increased throughout the experiment. The daily growth rate of fish in each treatment, varied. Fish that were fed with turmeric-enriched pellets showed a higher daily growth rate and as a consequence had a heavier BW than those with non-turmeric enriched pellets ( Table 3; Figure 2).

**Table 3.** Body weight of *Clarias batrachus* fed with turmeric enriched pellets and infected with *Aeromonas hydrophila* (on day 30).

Treatments	Body weight (gram) per week							Daily growth rate (g/day)
	D0	D7	D14	D21	D28	D35	D42	
P0	4.33	10.06	22.34	28.45	34.02	39.67	47.23	1.00
Pp	4.25	11.15	17.24	29.24	32.33	37.46	48.65	0.72
P1	4.47	12.67	20.56	28.12	35.56	40.56	50.37	1.09
P2	4.14	13.24	23.28	31.60	37.34	41.89	53.45	1.17
P3	4.24	14.14	24.22	32.21	38.23	44.43	55.23	1.21

Po, control (no turmeric/not infected); Pp, positive control (no turmeric/infected); P1, 0.5g turmeric in 1 Kg feed and infected; P2, 0.7g/Kg and infected; P3, 0.9g/Kg and infected.

As shown in Figure 2, the highest BW is in fish that are fed with feed pellets with 0.9 turmeric/Kg pellets. The lowest BW is in the fish that were not fed with turmeric-enriched pellets and infected with *A. hydrophila*. From the beginning (D0) to the end (D45) of the research, fish in all treatments grew well. Even after *A. hydrophila* infection, the growth of fish increased steadily. The growth of fish that are belonged to the Pp group had the lowest growth compared to growth of fish in other treatment groups.

### Hematological condition

In fish, the function of leucocytes is mainly related to the immune system. Feeding the fish with turmeric enriched pellets in this study aimed to improve the immunity of the fish. The immunity status of the fish was expected based on the leucocyte condition in general. The leucocyte condition of the fish before and after being fed with turmeric-enriched pellets is presented in Table 4.

**Table 4.** Leucocyte number in *Clarias batrachus* fed with turmeric enriched pellets and infected with *Aeromonas hydrophila* (on day 30).

Treatments	Leucocyte number (x10 ^4^ cells/mm ^3^)		
	D0	D30	D14inf
P0	8.18	8.37 ± 0.12 ^a^	8.59 ± 0.31 ^a^
Pp	8.26	8.47 ± 0.15 ^a^	9.71 ± 0.35 ^b^
P1	8.35	8.87 ± 0.2 ^b^	10.28 ± 0.31 ^b^
P2	8.43	9.34 ± 0.25 ^c^	11.08 ± 0.15 ^c^
P3	8.42	9.60 ± 0.15 ^c^	11.29 ± 0.45 ^c^

Po, control (no turmeric/not infected); Pp, positive control (no turmeric/infected); P1, 0.5g turmeric in 1 Kg feed and infected; P2, 0.7g/Kg and infected; P3, 0.9g/Kg and infected. Day14inf = 14 days after infection with *A. hydrophila.* Mean with standard error followed by different letters are significantly different (P<0.05)

Data obtained indicate that the number of leucocytes in the turmeric-fed fish and in the fish with no turmeric differs. The turmeric-fed fish showed a higher number of leucocytes (2–15×10 ^4^cells/mm ^3^). Before being feed with turmeric (day 0), the average number of leucocyte was 8.18–8.43×10 ^4^ cells/mm ^3^ and after being fed with turmeric enriched pellets for 30 days, the leucocytes slightly increased. P3 group fish has the highest number of leucocytes at day 30 (9.60×10 ^4^ cells/mm ^3^).

**Figure 3.**
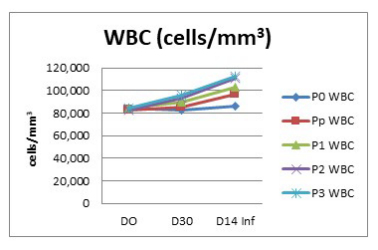
White blood cell count of *Claris batrachus* fed on turmeric-enriched pellets and infected with *Aeromonas hydrophila* (on day 30). Po, control (no turmeric/not infected); Pp, positive control (no turmeric/infected); P1, 0.5g turmeric in 1 Kg feed and infected; P2, 0.7g/Kg and infected; P3, 0.9g/Kg and infected. Day14inf = 14 days after infection with *A. hydrophila*.

After being infected, the number of leucocytes in the fish of each treatment increased due to infectious agents. The leucocyte of the P0 group is steady as the fish were not infected. The highest number of leucocytes was again seen in group P3 (11.29×10 ^4^cells/mm ^3^), while the lowest was in group Pp (9.71×10 ^4^cells/mm ^3^) ( Figure 3). These data suggest that the provision of turmeric increased the leucocyte number, before as well as after being infected with *A. hydrophila.*

**Figure 4.**
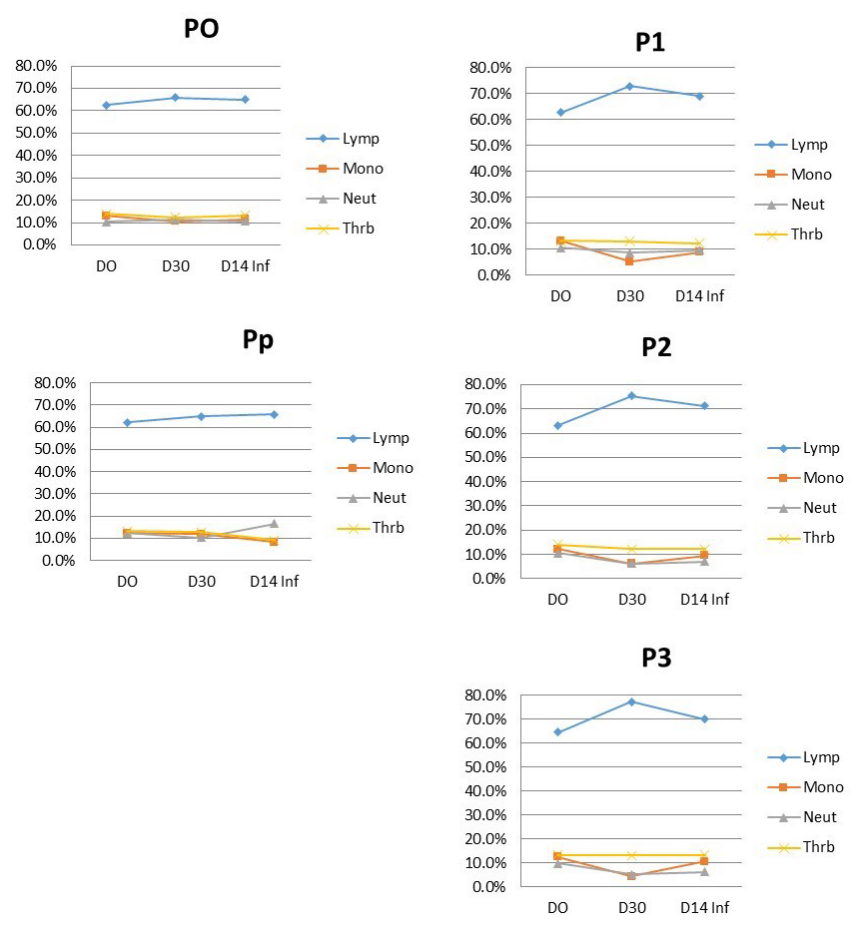
Leucocyte types in *Clarias batrachus* fed on turmeric-enriched pellets and infected with *Aeromonas hydrophila* (on day 30). P0, control (no turmeric/not infected); Pp, positive control (no turmeric/infected); P1, 0.5g turmeric in 1 Kg feed and infected; P2, 0.7g/Kg and infected; P3, 0.9g/Kg and infected. Day14inf = 14 days after infection with *A. hydrophila*.

The population of each leucocyte cell type in the treated fish are presented in Figure 4. The composition of leucocyte types in all treated fish showed a similar pattern. Lymphocytes were around 70% of the total population. From day 0 to the 30 ^th^ day, the fish were fed on turmeric and it is clear that in turmeric fed fish the lymphocyte proportion (>70%) is higher than fish not fed with turmeric. After being infected with *A. hydrophila*, the lymphocyte of the turmeric fed fish decreased slightly (lowest was 70%). On the other hand, the fish in group Pp, lymphocytes decrease moderately to approx. 65%. As the normal lymphocyte population in catfish is 71.66–73%, the leucocytes of the Pp group is relatively low and it means that the immunity of these fish is less able to combat the *A. hydrophila* infection.

## Discussion

In general, the survival of the fish treated with turmeric-enriched pellets in this study varied. Survival of the control positive fish (infected with *A. hydrophila* and no turmeric fed) was low (43.33%). The infected fish showed various clinical symptoms of MAS disease, namely ulcers, hemorrhage, pigmentation, swollen abdomen and eroded fins. Similar clinical signs have been found in common carp that suffer from MAS disease ^6– 8^. In addition, fish infected with 1.8×10 ^8^ CPU/ml of *A. hydrophila* die between 8 and 24 h and show alterations in behavior, which are not observed in control fish ^9, 10^.

In this study, the growth of fish that were fed with turmeric-enriched pellets was higher than the growth of the control positive fish. This suggests that turmeric improves the growth of fish, as shown by the increase in TL as well as BW. As the fish in the control positive group did not receive turmeric, their feeding appetite may be lower than the turmeric-fed fishes and this is reflected in their growth rate. The infection of *A. hydrophila* may worsen the health of the fish in general as their immunity is not being boosted by the turmeric and as a consequence, by the end of experiment, the BW of fish in Pp group is the lowest.

The fish that were treated with turmeric revealed better growth than that of the fish that do not receive any turmeric. This fact suggests that turmeric improves the growth of the fish. Curcumin supplementation has been shown to improve growth and feed appetite in *Nile tilapia* ^11^. Fish fed with feed enriched with curcumin exhibited enhanced antioxidant status and immune responses, and tilapia fed with curcumin supplemented diets had highest post-challenge survival rate ^12^. The higher curcumin content in fish feed resulted in a higher growth rate, as turmeric acts as a antibacterial, anti-inflammatory and antiviral agent ^13^. Curcumin has been shown to improve the immunity of fish and acts as a defense agent to combat the *A. hydrophila* infection ^14^. Curcumin is a strong antioxidant and acts as anti-free radical that negatively affects the physiological process of the fish ^2^. Turmeric contains curcumin, an active compound that is able to improve immunity as well as increases the appetite of the fish toward feed provided ^1^. As the turmeric-fed fish had a strong immunity and good appetite, they grew better than the fish with non-enriched pellets. In our study, after being infected on the 30 ^th^ day, the turmeric-fed fish was able to cope with the *A. hydrophila* attack and continue to grow.

In our study, after being infected, the number of leucocytes in the fish of each treatment group increased due to presence of an infectious agent. Among the infected fish, the increment of leucocyte number in the control positive group was the lowest, as most leucocytes are transferred to the infected area and leucocytes in blood vessels are reduced ^15^. On the other hand, the fish provided with turmeric had higher leucocyte numbers, which means that they are better at facing the infection, as leucocytes act as non-specific defense agents that are able to localize and eliminate pathogens. The immunogenic agents in natural remedies may trigger the increment of leucocytes in general ^15^. Increasing leucocyte numbers indicate that cellular immunity (non-specific immunity) of the fish is good ^16^.

Improvement of the immune system can be studied based on leucocyte cell type composition. In this study, the leucocytes of the control positive group was relatively low. A decrease of lymphocytes on the 14 ^th^ day after infection indicated that antibodies were formed to fight *A. hydrophila.* The fight may reduce lymphocyte cell numbers, as the lymphocyte in the peripheries are allocated to the infected area ^17^. Even though the lymphocytes in P1, P2 and P3 groups decreased after infection, their amount remained in the normal range. The ability of the fish to maintain the amount of lymphocytes may be caused by the presence of curcumin, which has been shown to trigger the formation of those cells ^18^. 

The presence of pathogens in the fish may trigger monocyte cells to regenerate. If the immunity of the fish is good, phagocytosis succeeds and the pathogen is defeated. The fish becomes healthy and the monocyte number increases to a normal range. The production of antibodies is crucial for the immune response ^12^. The monocyte percentage of *N. tilapia* fish ranges between 17 and 25% when present in freshwater ^12^. In less healthy fish, immunity is low, and monocyte action may not succeed. As result many monocyte cells die, the monocyte number decreases and the pathogen thrives. Monocytes or macrophages are able to phagocyte any pathogens, and if there is infection monocytes will move to the infected area ^19^. A decrease in monocytes may be caused by an increase in lymphocyte amount that produce antibodies; therefore leading to an obstruction of monocyte production. The pathogen present may disrupt the fish physiology and clinical signs of diseases occur. In this research, the clinical sign of MAS disease were present in the control positive group. This fact indicated that the fish with no turmeric fed had low immunity. In fish that were fed with turmeric (P1, P2 and P3) the number of monocytes was in the normal range. The condition of the fish in general showed few clinical symptoms of MAS disease. This fact indicates that the provision of turmeric is able to improve the immune system in fish, and fish in all treatment are able to defeat *A. hydrophila* infection.

Among the turmeric-fed fish, the P3 group showed the fewest clinical symptoms of MAS disease. This may be due to the best immune performance in P3 fish, as they had the highest number of lymphocytes (up to 80%). Based on data obtained, it can be concluded that the fish feed with 0.9g/Kg pellets provide the best result to improve fish immunity to fight *A. hydrophila* infection.

The lymphocyte level in this study ranged between 60–70%. The lymphocyte level of the freshwater fish ranged between 42 and 51%. The amount of lymphocytes could increase during stress. Stress in fish may interfere with non-specific immune responses, such as lymphocyte proliferation (increase in cell amount and alterations in T and B cells). Leukocyte increase is related to the decrease in cortisol levels in the body. When cortisol level decreased, DNA synthesis of the lymphocyte cells occurred and led to the high amount of lymphocytes ^20^.

## Conclusions

Feeding *C. batrachus* with turmeric-enriched pellets is effective in improving the immune system of the fish. The number of lymphocytes maintained a normal range even though the fish were infected with *A. hydrophila*. The clinical signs of MAS disease were fewer lighter in fish that were fed with turmeric, and the most effective dose of turmeric for improving the immune system of the fish was shown to be 0.9g/Kg of pellets.

## Data availability

### Underlying data

Figshare: Survival Rate, TL and Growth, https://doi.org/10.6084/m9.figshare.13606265.v1 ^21^.

This project contains the following underlying data:

- Survival rates for all aquaria (n=15),
- Clinical signs of MAS for all aquaria (n=15),
- Body weight of all fish (n=15),
- Total length of all fish (n=15),
- Number of leucocytes in all fish (n=15),
- Number of lymphocytes, monocytes, neutrophils, thrombocytes in all fish (n=15).

Data are available under the terms of the Creative Commons Attribution 4.0 International license (CC-BY 4.0).
