# Supplementary material for: Effectiveness of turmeric-enriched pellets to improve the immunity of Clarias batrachus toward motile Aeromonas septicemia disease
Source: F1000Res. 2021 May 17;10:169. Originally published 2021 Mar 2. [Version 2] doi: 10.12688/f1000research.28260.2 (PMC8191517; doi:10.12688/f1000research.28260.2)
Supplement: Peer review history

# Peer review: Reviewer response for version 2

**DOI:** 10.5256/f1000research.56750.r85485

**Published:** 2021-06-09

**Authors:** Indra Junaidi Zakaria

**Affiliations:** 1 Department of Biology, Universitas Andalas, Padang, Indonesia

Okay.

Is the work clearly and accurately presented and does it cite the current literature?

Partly

If applicable, is the statistical analysis and its interpretation appropriate?

Partly

Are all the source data underlying the results available to ensure full reproducibility?

Partly

Is the study design appropriate and is the work technically sound?

No

Are the conclusions drawn adequately supported by the results?

Partly

Are sufficient details of methods and analysis provided to allow replication by others?

Yes

Reviewer Expertise:

Aquaculture (marine and freshwater), Ichthyology, Fish Biology, Marine Biology

I confirm that I have read this submission and believe that I have an appropriate level of expertise to confirm that it is of an acceptable scientific standard.

# Peer review: Reviewer response for version 2

**DOI:** 10.5256/f1000research.56750.r85484

**Published:** 2021-06-09

**Authors:** Christopher Marlowe A. Caipang

**Affiliations:** 1 College of Liberal Arts, Sciences, and Education, University of San Agustin, Iloilo City, Philippines

The authors have addressed the comments and suggestions that were made in the original submission.

Is the work clearly and accurately presented and does it cite the current literature?

Partly

If applicable, is the statistical analysis and its interpretation appropriate?

Partly

Are all the source data underlying the results available to ensure full reproducibility?

Partly

Is the study design appropriate and is the work technically sound?

Partly

Are the conclusions drawn adequately supported by the results?

Partly

Are sufficient details of methods and analysis provided to allow replication by others?

Partly

Reviewer Expertise:

fish health management, aquatic biotechnology

I confirm that I have read this submission and believe that I have an appropriate level of expertise to confirm that it is of an acceptable scientific standard.

# Peer review: Reviewer response for version 1

**DOI:** 10.5256/f1000research.31256.r80815

**Published:** 2021-03-22

**Authors:** Indra Junaidi Zakaria

**Affiliations:** 1 Department of Biology, Universitas Andalas, Padang, Indonesia

- What about the influence of the water qualities in which the fish were kept during the experiment? Such as temperature, pH, DO, BOD, ammonia and phosphate and otherwise; is there no data? The health of the fish during cultivation also depends on the water quality. Or you should make the statement that the aquatic environment has no effect. 
- On the tree treatment of adding turmeric to the pellets, it was seen that up to the day of observation, the movement of the graph was almost the same, which was the same as upward. Will this be valid for how many days after that?

Is the work clearly and accurately presented and does it cite the current literature?

Partly

If applicable, is the statistical analysis and its interpretation appropriate?

Partly

Are all the source data underlying the results available to ensure full reproducibility?

Partly

Is the study design appropriate and is the work technically sound?

No

Are the conclusions drawn adequately supported by the results?

Partly

Are sufficient details of methods and analysis provided to allow replication by others?

Yes

Reviewer Expertise:

Aquaculture (marine and freshwater), Ichthyology, Fish Biology, Marine Biology

I confirm that I have read this submission and believe that I have an appropriate level of expertise to confirm that it is of an acceptable scientific standard, however I have significant reservations, as outlined above.

# Response

**Published:** 2021-04-27

**Authors:** Morina Riauwaty

**Affiliations:** Universitas Riau, Indonesia

- What about the influence of the water qualities in which the fish were kept during the experiment? Such as temperature, pH, DO, BOD, ammonia and phosphate and otherwise; is there no data? The health of the fish during cultivation also depends on the water quality. Or you should make the statement that the aquatic environment has no effect. 

*OK, the water quality data has been provided and the relation between water quality and the fish health has been explained.*

- On the tree treatment of adding turmeric to the pellets, it was seen that up to the day of observation, the movement of the graph was almost the same, which was the same as upward. Will this be valid for how many days after that?

*I have explained that the growth rate of the turmeric feeding fish is higher and the graph is valid as long as the fish is fed with turmeric enriched pellets.*

# Peer review: Reviewer response for version 1

**DOI:** 10.5256/f1000research.31256.r80812

**Published:** 2021-03-19

**Authors:** Christopher Marlowe A. Caipang

**Affiliations:** 1 College of Liberal Arts, Sciences, and Education, University of San Agustin, Iloilo City, Philippines

The present study describes the use of turmeric in improving immunity of Clarias batrachus against infection with MAS. Results showed incorporation of 9g turmeric powder per kilogram of feeds improved immunity of the fish.

 The results presented are straightforward. However, the manuscript needs improvement on the following aspects, which I hope the authors would consider:

1. The authors mentioned that turmeric causes stress when fish are immersed in a solution containing that rhizome. Please cite studies that turmeric is beneficial to fish when incorporated in the diets.
2. The authors claimed that feeding fish with turmeric-enriched diets has not been studied. I suggest that the authors make a thorough literature search to indeed claim that no studies have done so.
3. In the preparation of the enriched diets, there was no mention how turmeric powder was incorporated in the feed pellets. Based from the manuscript, the turmeric powder was just mixed with the feed pellets. If this the case, upon feeding the turmeric powder would immediately leach out from the feeds and thus may not be consumed by the fish.
4. For the blood sampling, only 3 fish per treatment was used and there were 3 replicate tanks per treatment with 10 fish in each replicate. So this means that only 1 fish was taken per replicate tank to make up for 3 fish/treatment? Please explain.
5. Were the same fish used for blood sampling at initial, day 30 of feeding and at 14th day of infection?
6. Please indicate the statistical tool that was used.
7. As all fish survived the challenge, then there is no need for Table 1. The authors can mention in the manuscript that all fish survived the experimental infection.
8. I suggest to combine Table 2 and Fig 1, as well as Table 3 and Fig 2 as these provide same information.
9. If the authors can show photos of the WBC for catfish that would add more strength to the manuscript, as the morphology of leukocytes in fish varies. This would be helpful to other researchers who would be working similar studies in the future as they can refer to the photos of the different leukocytes from this manuscript.
10. I suggest to revise Fig 4 to make the data presentation clear. The trend of each leukocyte type in the different treatments must be compared statistically rather than presenting the leukocyte populations of every treatment as what the authors presented in the present manuscript.

I hope that the authors will incorporate these comments/suggestions when they revise their manuscript.

Is the work clearly and accurately presented and does it cite the current literature?

Partly

If applicable, is the statistical analysis and its interpretation appropriate?

Partly

Are all the source data underlying the results available to ensure full reproducibility?

Partly

Is the study design appropriate and is the work technically sound?

Partly

Are the conclusions drawn adequately supported by the results?

Partly

Are sufficient details of methods and analysis provided to allow replication by others?

Partly

Reviewer Expertise:

fish health management, aquatic biotechnology

I confirm that I have read this submission and believe that I have an appropriate level of expertise to confirm that it is of an acceptable scientific standard, however I have significant reservations, as outlined above.

# Response

**Published:** 2021-04-27

**Authors:** Morina Riauwaty

**Affiliations:** Universitas Riau, Indonesia

The present study describes the use of turmeric in improving immunity of Clarias batrachus against infection with MAS. Results showed incorporation of 9 g turmeric powder per kilogram of feeds improved immunity of the fish.

 The results presented are straightforward. However, the manuscript needs improvement on the following aspects, which I hope the authors would consider:

 1. The authors mentioned that turmeric causes stress when fish are immersed in a solution containing that rhizome. Please cite studies that turmeric is beneficial to fish when incorporated in the diets.

*I have added the literature on the turmeric beneficial in culturing the fish.*

 2. The authors claimed that feeding fish with turmeric-enriched diets has not been studied. I suggest that the authors make a thorough literature search to indeed claim that no studies have done so.

*I have added the literature on the use of turmeric for feeding fish.*

 3. In the preparation of the enriched diets, there was no mention how turmeric powder was incorporated in the feed pellets. Based from the manuscript, the turmeric powder was just mixed with the feed pellets. If this the case, upon feeding the turmeric powder would immediately leach out from the feeds and thus may not be consumed by the fish.

*I have explained that the turmeric powder was mixed with water and the powder was stuck in the pellet granule. The powder was swallowed when the fish was eating the pellets.*

 4. For the blood sampling, only 3 fish per treatment was used and there were 3 replicate tanks per treatment with 10 fish in each replicate. So this means that only 1 fish was taken per replicate tank to make up for 3 fish/treatment? Please explain.

*I have added information on the blood sampling. In this research there were 5 treatments and in each treatment there were 3 replications (in 3 aquaria). For blood sampling, 3 fishes were taken from each aquarium, it means that there were 9 fishes/ treatment or total 45 fishes were used for blood study.*

 5. Were the same fish used for blood sampling at initial, day 30 of feeding and at 14th day of infection?

*The fish for blood sampling were returned to each aquarium and for the next sampling the fish was taken randomly. It means that the sampled fish might be accidentally captured for the next samplings.*

 6. Please indicate the statistical tool that was used.

*I have added the statistical tool that was used.*

 7. As all fish survived the challenge, then there is no need for Table 1. The authors can mention in the manuscript that all fish survived the experimental infection.

*I have removed Table 1 and explained the survival information in manuscript.*

 8.Suggest to combine Table 2 and Fig 1, as well as Table 3 and Fig 2 as these provide same information.

*OK, I have combined Table 2 and Fig 1, as well as Table 3 and Fig 2.*

 9.If the authors can show photos of the WBC for catfish that would add more strength to the manuscript, as the morphology of leukocytes in fish varies. This would be helpful to other researchers who would be working similar studies in the future as they can refer to the photos of the different leukocytes from this manuscript.

*I have added the photo of the WBC of catfish.*

 10. I suggest to revise Fig 4 to make the data presentation clear. The trend of each leukocyte type in the different treatments must be compared statistically rather than presenting the leukocyte populations of every treatment as what the authors presented in the present manuscript.

*OK, I have revised Fig 4.*
